# Revisiting the Status of Yellow Fever Epizootics and Its Surveillance in South America: New Non-Human Primates, Spillover and Ecological Drivers

**DOI:** 10.3390/pathogens15040412

**Published:** 2026-04-10

**Authors:** D. Katterine Bonilla-Aldana, Jorge Luis Bonilla-Aldana, Lysien Zambrano, Alfonso J. Rodriguez-Morales

**Affiliations:** 1 College of Medicine, Korea University, Seoul 02841, Republic of Korea; dbonilla@korea.ac.kr; 2 Grupo de Virologia, Universidad El Bosque, Bogotá 111321, DC, Colombia; jlbonilla@unbosque.edu.co; 3 Department of Morphological Sciences, School of Medical Sciences, Universidad Nacional Autónoma de Honduras, Tegucigalpa 11101, Honduras; lysien.zambrano@unah.edu.hn; 4 Faculty of Health Sciences, Universidad Científica del Sur, Lima 15046, Peru; 5 Grupo de Investigación Biomedicina, Faculty of Medicine, Fundación Universitaria Autónoma de las Américas-Institución Universitaria Visión de las Américas, Pereira 660003, Colombia

**Keywords:** yellow fever, epizootics, non-human primates, surveillance, spillover, one health, South America

## Abstract

Yellow fever (YF) remains a re-emerging vector-borne zoonotic disease in tropical regions of the Americas despite the availability of an effective vaccine. In South America, the virus is maintained through a jungle transmission cycle involving *Haemagogus* and *Sabethes* mosquitoes and non-human primates (NHPs), which act as amplifying hosts and key epidemiological sentinels. This narrative review examines the current status of YF epizootics in South America, with a focus on the role of NHPs in viral circulation, early detection, and spillover risk to human populations. We synthesize recent evidence on epizootic patterns across endemic countries, the differential susceptibility of neotropical primates, and the ecological and environmental drivers influencing transmission, including deforestation, habitat fragmentation, and human encroachment into forested areas. In addition, we analyze current surveillance strategies, including wildlife monitoring, entomological and genomic surveillance, and their integration within a One Health framework. This review highlights that YF epizootics are expanding geographically and are closely linked to environmental change and human–ecosystem interactions. Strengthening integrated, multidisciplinary surveillance systems is essential to improve early detection, guide vaccination strategies, and prevent human outbreaks. These findings underscore the critical importance of operationalizing the One Health approach to enhance preparedness and response to YF in South America.

## 1. Introduction

Yellow fever (YF) is an acute viral hemorrhagic disease caused by the YF virus, a mosquito-borne Flavivirus (*Orthoflavivirus flavi*), which continues to represent a major public health problem in tropical regions of Africa and the Americas [1,2,3]. Despite the availability of a highly effective vaccine for more than eight decades [4], the disease persists due to complex interactions among ecological, epidemiological, and social factors that favor viral circulation in jungle ecosystems [5]. In South America, transmission is mainly maintained through the jungle cycle, in which mosquitoes of the genera *Haemagogus* and *Sabethes* act as vectors, while various non-human primates (NHPs) function as amplifying hosts and epidemiological sentinels of the virus [6]. In this context, surveillance of epizootics in NHPs has become a fundamental tool for early detection of the risk of transmission to humans and for guiding prevention strategies [3,6,7,8].

NHPs play a key role in the ecology of the YF virus in the Americas [6]. Several neotropical species, especially those belonging to the families Atelidae (such as howler monkeys of the genus *Alouatta*) and Cebidae, are highly susceptible to infection and may experience high case fatality rates during epizootics (Table 1) [9,10].

**Figure 1:**
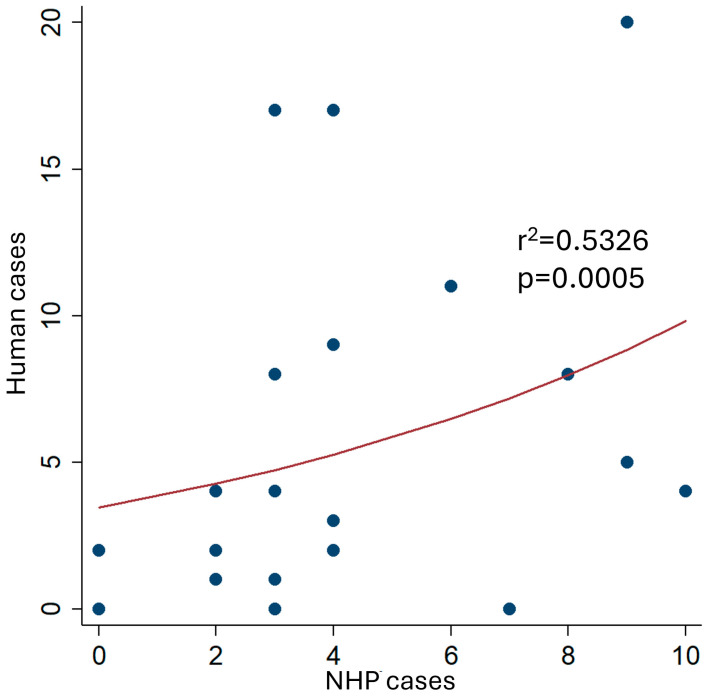
Relationship between non-human primate (NHP) cases and human yellow fever cases during the early phase of the 2024/2025 outbreak in Brazil (epidemiological weeks 50–2024 to 19–2025). The analysis includes 126 human cases and 95 NHPs cases reported during the study period, based on official surveillance data from the Brazilian Ministry of Health.

This susceptibility makes these species early indicators of viral circulation in jungle areas [6]. In multiple South American countries, including Brazil, Colombia, Peru, and Bolivia, reports of mortality in NHPs have historically preceded the appearance of human cases, underscoring the value of these animals as early warning systems within epidemiological surveillance programs [11,12,13]. Therefore, the systematic notification and analysis of epizootics in NHPs is an essential component to anticipate human outbreaks and activate control measures, such as vaccination campaigns in at-risk populations [14]. For example, in Brazil, the number of YF cases among NHPs was significantly associated with the number of human YF cases during the early phase of the 2024/2025 outbreak (Figure 1). This correlation underscores the pivotal role of NHPS as early epidemiological sentinels of yellow fever virus circulation in sylvatic ecosystems. Notably, increases in NHPs mortality frequently precede the detection of human cases, indicating ongoing viral amplification in the jungle transmission cycle. This temporal and epidemiological relationship reinforces the importance of integrating wildlife surveillance into public health systems. Early detection of epizootics in primates can trigger proactive interventions, including targeted vaccination, enhanced entomological surveillance, and community-based risk mitigation, thereby reducing the likelihood and magnitude of human outbreaks (https://www.gov.br/saude/pt-br/composicao/svsa/cnie/painel-febre-amarela) (accessed on 1 April 2026) (Figure 1).

From a zoonotic perspective, YF is a clear example of an emerging disease maintained by complex ecological cycles involving interactions among wildlife, vectors, and humans [11,12,13]. Although humans are not the natural reservoir of the virus in the American jungle cycle, they can serve as accidental hosts when entering ecosystems where the virus is actively circulating [7]. This phenomenon is particularly relevant in the context of environmental changes, deforestation, agricultural expansion, urbanization, and human mobility into forest areas, factors that increase opportunities for contact among humans, vectors, and infected primates [15]. Likewise, the presence of urban mosquitoes such as *Aedes aegypti* poses the potential risk of reurbanization of the transmission cycle, which could facilitate the spread of the virus in densely populated areas if introduced from jungle environments [16].

In this scenario, the One Health approach is becoming increasingly important for understanding and addressing the dynamics of YF [5,17]. This approach recognizes that human, animal, and environmental health are deeply interconnected. In the case of YF, effective surveillance requires integrating data from different disciplines, including human epidemiology, veterinary medicine, vector ecology, biodiversity conservation, and environmental monitoring [18,19]. The detection of epizootics in primates, the analysis of mosquito vector populations, and the evaluation of ecological changes in forest ecosystems enable us to generate a comprehensive view of transmission risk [6]. In this way, the implementation of integrated surveillance systems facilitates the early identification of risk areas and contributes to timely decision-making in public health [20].

While multiple surveillance approaches are currently implemented, each presents specific strengths and limitations. Passive surveillance based on reporting of non-human primate mortality is highly valuable as an early warning system, particularly in remote or forested areas; however, it is dependent on timely reporting and may be affected by under-detection. Laboratory-based confirmation, including reverse transcription polymerase chain reaction (RT-PCR) and histopathology, provides high diagnostic accuracy but requires technical capacity and may delay response in resource-limited settings. Entomological surveillance contributes to understanding vector distribution and transmission risk, although it is logistically demanding and often limited in spatial coverage. More recently, genomic surveillance has enhanced the ability to track viral dispersion and evolution, but its implementation remains uneven across the region. No single approach is sufficient on its own; rather, their integration is essential. However, operationalizing this integration within a One Health framework remains challenging due to fragmentation between human, animal, and environmental health sectors, differences in surveillance capacities across countries, and limitations in data sharing and coordination. Strengthening intersectoral collaboration and harmonizing surveillance systems are therefore critical to improving early detection and response to YF epizootics [21,22].

Surveillance of epizootics in NHPs has been strengthened in South America in recent decades, particularly following large outbreaks in Brazil between 2016 and 2018, which highlighted the magnitude of the disease’s ecological and health impacts [23,24]. During these events, thousands of primates died as a result of the infection, which generated a regional alert about the need to improve monitoring, diagnosis, and notification systems [6]. Currently, several countries have adopted specific protocols for mortality investigations in NHPs, including sample collection, diagnostic confirmation using molecular or histopathological techniques, and geospatial analysis of events [17]. These systems enable the identification of active viral foci and the targeting of preventive interventions to nearby human populations [6].

The prevention of YF in humans depends fundamentally on vaccination, which is considered one of the most effective control measures [25]. However, vaccination coverage remains heterogeneous across several regions of South America, thereby maintaining susceptible populations to the introduction of the virus from jungle cycles. In this sense, the early detection of epizootics in primates offers a critical opportunity to implement preventive vaccination strategies, strengthen entomological surveillance, and promote educational interventions aimed at exposed communities [24]. In addition, the protection of NHPs also has important implications for biodiversity conservation, given that some highly susceptible species may experience significant population declines during outbreaks [26].

Overall, understanding the dynamics of YF epizootics in NHPs and their relationship to ecological and epidemiological factors is essential to strengthen regional surveillance and prevent transmission to humans [17]. This narrative review examines the current state of knowledge on YF epizootics in South America, with particular emphasis on the roles of NHPs as epidemiological sentinels, zoonotic transmission mechanisms, ecological factors that modulate viral circulation, and surveillance and prevention strategies from a One Health perspective. The analysis of these elements seeks to contribute to a better understanding of current challenges and to the formulation of integrated strategies for the control and prevention of this re-emerging disease in the region [17].

By synthesizing current evidence and identifying key knowledge gaps, this review aims to inform clinicians, researchers, and policymakers and to support the development of more effective surveillance strategies and prevention measures for yellow fever (YF) in South America. To achieve this, we conducted a structured literature search across multiple databases, including Web of Science, Scopus, PubMed, SciELO, LILACS, Latindex, and ScienceDirect. Additionally, we were supported by evidence assessment tools such as OpenEvidence and VeraHealth platforms; however, this work does not constitute a systematic or scoping review. The search strategy was designed to ensure both breadth and specificity in accordance with the objectives of this narrative review. Search terms included combinations of “Yellow fever”, “YF epizootics”, “non-human primates”, “surveillance”, “spillover”, “One Health”, “ecological drivers”, and “South America”. Eligible studies were those presenting original data or relevant epidemiological, ecological, or surveillance insights. Articles were excluded if they contained incomplete or overlapping datasets or lacked access to full text. These criteria were applied to maintain methodological rigor and ensure the validity and relevance of the synthesized evidence.

## 2. YF Virus Overview

YF is an acute viral hemorrhagic disease caused by the YF virus (YFV), *Orthoflavivirus flavi,* belonging to the genus *Flavivirus* of the family *Flaviviridae* [27]. It is transmitted mainly by hematophagous mosquitoes and is maintained in nature through transmission cycles involving vectors and vertebrate hosts [28]. There are three recognized epidemiological cycles: the jungle (or wild) cycle, the intermediate cycle (mainly described in Africa), and the urban cycle [29]. In South America, the jungle cycle predominates, where the virus circulates among mosquitoes of the genera *Haemagogus* and *Sabethes* and NHP, while humans are incidentally infected when entering forest environments where the virus is active [16,30]. Clinically, the disease can range from mild and self-limiting forms to severe symptoms characterized by fever, jaundice, hemorrhages, and multiorgan failure, with case fatality rates that can reach between 20% and 50% in severe cases [31]. Although there is a highly effective vaccine based on the attenuated 17D virus, YF continues to cause periodic outbreaks in endemic regions, particularly in tropical areas of Africa and South America, where the interaction between ecological factors, vector density, non-human primate susceptibility, and human vaccine coverage conditions the dynamics of virus transmission [32]. In this context, the disease represents a clear example of a vector-borne zoonosis whose persistence is closely linked to the ecology of tropical ecosystems [33].

## 3. YF in NHPs

YFV plays a central role in the ecological dynamics of YF in South America, where NHPS constitute one of the main vertebrate hosts in the jungle cycle of transmission [7]. These animals actively participate in viral amplification and, at the same time, serve as epidemiological indicators of viral circulation in tropical ecosystems [6]. The interaction between the virus, susceptible primates, and mosquito vectors establishes a complex ecological system that maintains the persistence of the virus in nature and determines the risk of transmission to human populations [7,34,35].

In the Americas, the YF jungle cycle is characterized by the circulation of YFV among forest-dwelling mosquitoes, mainly of the genera *Haemagogus* and *Sabethes*, and among neotropical primates [36]. When an infected mosquito feeds on the blood of a susceptible primate, the virus can replicate in the vertebrate host and reach viremia levels sufficient to infect new vectors during subsequent bites. This process allows the cycle to be maintained in nature without human intervention. However, when people enter jungle environments where the virus is actively circulating, they can be bitten by infected mosquitoes and become incidental hosts [37,38,39].

Neotropical primates show variable susceptibility to YFV infection, depending on the species. In general, howler monkeys of the genus *Alouatta* (family Atelidae) are considered the most susceptible and have high mortality rates during epizootics. These species can experience fulminant infections with severe liver damage, similar to those seen in humans, which frequently leads to death within a few days (Table 2) [33,40]. Due to this high sensitivity to the virus, howler monkeys have been recognized as important natural sentinels of viral circulation in South America. Detection of mortality in these populations often precedes the appearance of human cases, providing an early signal of viral activity in a given region [40].

Other primate species, such as capuchin monkeys (*Sapajus* and *Zebus*) and squirrel monkeys (*Saimiri*), can also be infected with the YF virus. However, they generally have lower mortality and fewer subclinical infections than *Alouatta*. This variability in response to infection suggests differences in immunological susceptibility between species and possible evolutionary adaptations to the virus’s historical circulation in Neotropical ecosystems. In some cases, primates can survive infection and develop neutralizing antibodies, which contribute to the immune dynamics of wild populations [9,33,41].

Table 2 summarizes key differences in the clinical presentation and severity of yellow fever infection between New World and Old World NHP. Overall, New World primates show markedly higher susceptibility to severe disease, characterized by rapid clinical deterioration, extensive hepatic damage, and high mortality during epizootics. In contrast, Old World primates generally exhibit milder or subclinical infections, with lower mortality and limited systemic involvement under natural conditions. These differences are consistent with the hypothesis of a longer evolutionary coexistence between the yellow fever virus and African primates, which may confer partial resistance or tolerance to infection.

From an epidemiological perspective, these contrasting patterns are highly relevant. The high lethality observed in New World primates, particularly in genera such as *Alouatta*, makes them highly sensitive indicators of viral circulation and effective early warning sentinels. Conversely, the lower clinical impact in Old World primates suggests a different role in transmission dynamics, potentially contributing to viral maintenance without producing conspicuous mortality signals. Thus, the information presented in Table 2 highlights the importance of host-specific responses in shaping both the clinical expression of the disease and its utility for surveillance within a One Health framework. These distinctions should be considered when designing surveillance and risk assessment strategies across different geographic regions.

From a pathogenic perspective, YFV exhibits a particular tropism for the liver, where it replicates intensively, leading to hepatocellular necrosis and liver dysfunction (Table 2). In highly susceptible NHP, pathological findings include hepatocellular degeneration, mediozonal or panlobular necrosis, an inflammatory infiltrate, and Councilman bodies, which are characteristic of virus-induced hepatocellular apoptosis [42]. In addition to liver damage, alterations in other organs, such as the spleen, kidneys, and lymph nodes, may also be observed, reflecting the systemic nature of the infection [43,44]. These pathological changes are comparable to those observed in humans with severe YF, which has allowed some primates to be used as experimental models for the study of the disease [45].

**Figure 2:**
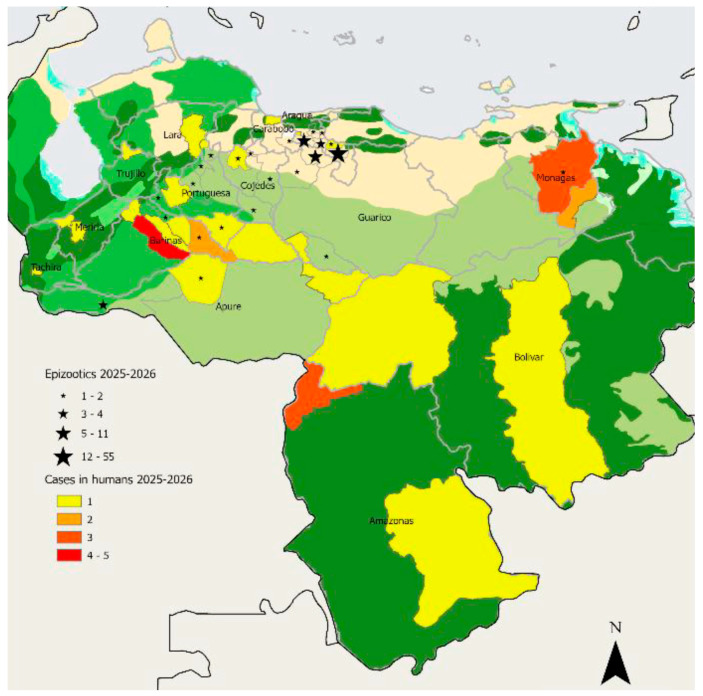
Confirmed and suspected yellow fever cases in humans and epizootics in NHPs by state in Venezuela, 2025–2026 (up to epidemiological week 7 of 2026). The analysis includes 39 confirmed human cases and 109 NHPS epizootic events reported during the study period, based on data from the Pan American Health Organization (PAHO) (https://www.paho.org/en/documents/epidemiological-alert-yellow-fever-americas-region-13-march-2026) (accessed on 1 April 2026).

YF epizootics in NHPs are epidemiological events of great importance for public health. During these episodes, a significant increase in mortality is observed in primate populations, particularly in highly susceptible species. In South America, epizootics have been documented in numerous countries, including Brazil, Colombia, Peru, Bolivia, and Venezuela (Table 3) [6]. In many cases, these events have preceded human outbreaks temporally and geographically, which has made it possible to identify areas of active transmission and activate control strategies [44,46]. With the recent report of YF in Venezuela, the Pan American Health Organization (PAHO) has also provided information on the epizootics in that country (https://www.paho.org/en/documents/epidemiological-alert-yellow-fever-americas-region-13-march-2026) (accessed on 1 April 2026). During 2025, up to epidemiological week (EW) 53, a total of 90 epizootics of yellow fever in NHPs were reported in the following states: Apure (*n* = 3), Aragua (*n* = 66), Barinas (*n* = 2), Carabobo (*n* = 2), Cojedes (*n* = 2), Guárico (*n* = 7), Lara (*n* = 1), Portuguesa (*n* = 6), and Monagas (*n* = 1) (Figure 2). Of these, eight epizootics were laboratory confirmed. In 2026, up to EW 9, 19 suspected epizootics in NHPs had been reported in the states of Aragua (*n* = 8), Cojedes (*n* = 5), Apure (*n* = 2), and Guárico (*n* = 4); however, none had yet been confirmed by laboratory testing (Figure 2).

The data presented in Table 3 reveal several important epidemiological patterns. First, a marked concentration of epizootics is observed in Brazil and Venezuela, which together account for the majority of reported events, suggesting these countries remain key hotspots of YF viral circulation in the region. Within Brazil, states such as São Paulo and Minas Gerais show a particularly high number of epizootics, reflecting sustained transmission in areas characterized by fragmented forest landscapes and close human–wildlife interfaces. Similarly, in Venezuela, the high number of events in Aragua and neighboring states indicates active viral circulation and potential expansion into new areas. In Colombia, clusters in departments such as Tolima and Putumayo highlight areas of ongoing risk, particularly in regions undergoing ecological change and land-use modification.

These patterns suggest that epizootics are not randomly distributed but are strongly influenced by ecological and environmental factors. Areas with intense deforestation, habitat fragmentation, and increased human activity tend to facilitate contact between vectors, NHP, and human populations, thereby increasing the likelihood of viral amplification and spillover. Additionally, the distribution of competent mosquito vectors and the presence of highly susceptible primate species further shape the spatial dynamics of outbreaks. Although direct correlations cannot be established in this dataset, the observed trends are consistent with a One Health framework, in which environmental change, biodiversity, and vector ecology interact to drive YF transmission. These findings underscore the importance of targeted surveillance and preventive strategies in identified high-risk areas.

One of the most relevant examples occurred in Brazil between 2016 and 2018, when one of the largest YF epizootics in the continent’s recent history was recorded. During this period, thousands of primates died as a result of the infection, particularly howler monkeys, which evidenced the intense circulation of the virus in wide areas of the country. This event highlighted the importance of primate mortality surveillance systems for early detection of viral activity and for the implementation of vaccination campaigns in at-risk human populations [48].

Surveillance of YF in NHPs is based on the detection, reporting, and investigation of mortality or disease events in these animals. In many countries, surveillance programs involve local communities, park rangers, environmental authorities, and public health personnel, who report the presence of dead or sick primates. Once a suspicious event has been detected, biological samples are collected to confirm the infection using laboratory methods, such as RT-PCR, viral isolation, or histopathological studies with immunohistochemical techniques [49].

The spatial analysis of epizootics also enables the identification of patterns of viral dispersion over time and space. In general, the spread of YFV in jungle environments may follow ecological corridors associated with primate and vector distribution, as well as with landscape features such as forest fragmentation, habitat connectivity, and the presence of riparian corridors. These ecological factors influence transmission dynamics and can facilitate the virus’s spread to new areas [50].

From a conservation perspective, YF epizootics also pose a significant threat to some Neotropical primate species. The high mortality observed in highly susceptible species can lead to significant population declines, especially in areas already affected by habitat loss, hunting, or ecosystem fragmentation. In this sense, YF is not only a public health problem, but also a challenge for the conservation of biodiversity in South America [42].

The integration of non-human primate surveillance within public health strategies reflects the growing recognition of the One Health approach, which promotes collaboration across disciplines to address zoonotic diseases at the interface between humans, animals, and ecosystems. In the case of YF, monitoring epizootics in primates provides critical information on the virus’s circulation and allows us to anticipate risks to human populations. This information is critical to guide preventive interventions, such as vaccination campaigns, entomological surveillance, and community education [42].

## 4. Clinical Findings in New and Old World NHPs

Clinical findings of YFV infection in NHPs vary widely across species and geographic regions, particularly among New World (Americas) and Old World (Africa and Asia) primates (Table 2) [33]. In Neotropical primates, many species are highly susceptible to the virus and can develop severe disease with high mortality during epizootics. In contrast, several Old World primates appear to have greater relative resistance to natural infection, suggesting a longer coevolution between the virus and its hosts in Africa. These biological differences significantly influence the clinical presentation of the disease and the epidemiological role of primates within transmission cycles (Table 2) [33].

In New World primates, especially in highly susceptible species such as howler monkeys (*Alouatta*) (Table 1), the infection usually manifests as an acute and rapidly progressive disease. Infected animals may present with initial nonspecific clinical signs such as lethargy, weakness, decreased activity, and loss of appetite [10]. As the infection progresses, more severe manifestations may be observed, including dehydration, prostration, difficulty moving around, and, in some cases, neurological signs such as tremors or disorientation. In many epizootic episodes, the clinical course is so rapid that the animals are found dead without having been previously observed with obvious symptoms. High viremia and intense viral replication in key organs, particularly the liver, contribute to the rapid clinical deterioration [51].

In addition to the general signs, some infected primates may present clinical manifestations consistent with hepatic and systemic involvement, similar to those observed in humans with severe YF (Table 2). These include jaundice, marked weakness, and bleeding abnormalities, although in most cases, the clinical findings observable in the field are limited due to the rapid progression of the disease [42]. Experimental and observational studies have shown that infection in susceptible primates results in intense hepatocellular necrosis, leading to acute liver failure. Alterations in other organs, such as the spleen and kidneys, may also be observed, reflecting the systemic nature of the infection. These pathological features explain the high mortality observed in some Neotropical primate species during outbreaks [9].

In contrast, Old World primates, such as several African species of the genus *Cercopithecus* or *Papio*, tend to present milder or even subclinical infections under natural conditions (Table 2). Although they can develop viremia sufficient to infect mosquito vectors, severe clinical disease is less common than in New World primates. This lower susceptibility has been interpreted as resulting from a longer evolutionary relationship between the YF virus and African primates, which would have favored the selection of mechanisms of resistance or tolerance to infection [52]. However, under experimental conditions, some species may develop systemic disease with clinical signs comparable to those observed in Neotropical primates. Taken together, these clinical and epidemiological differences between New and Old World primates highlight the importance of considering host biodiversity in the study of YF and in the design of surveillance and prevention strategies [33].

## 5. Epizootics in the Last Decade

Over the last decades, YF epizootics in NHPs have gained increasing epidemiological relevance in South America, reflecting changes in the dynamics of virus transmission in tropical ecosystems (Table 3) [17,23,33]. Since the late twentieth and early twenty-first centuries, several countries in the region have documented recurrent episodes of mass mortality in primate populations, particularly in Amazonian areas and tropical forests. These events have been reported in Brazil, Colombia, Peru, Bolivia, and Venezuela, where the virus circulates through the jungle cycle between mosquitoes of the genera Haemagogus and Sabethes and various species of primates [39,53]. In many cases, primate epizootics have preceded the emergence of human cases, cementing their value as early indicators of viral activity and key tools for epidemiological surveillance. Routine reporting of mortality in NHPs has thus become an essential component of YF surveillance programs in the region [23].

One of the most significant events in recent decades occurred in Brazil between 2016 and 2018, when the largest YF epizootic in South America was documented. During this period, the virus spread from Amazonian areas to regions of southeastern Brazil that had historically had low disease activity [54]. The death of thousands of primates accompanied this process, mainly howler monkeys of the genus *Alouatta*, which are highly susceptible to infection. The magnitude of this epizootic demonstrated the virus’s ability to move along ecological corridors and forest fragments, facilitated by the mobility of vectors and the distribution of primate hosts. In addition, this event highlighted the importance of integrated surveillance systems that combine the detection of epizootics, entomological monitoring, and preventive vaccination in human populations at risk [10,55].

Significant epizootics have also been recorded in other South American countries in recent decades, confirming the virus’s persistence in jungle ecosystems and its potential for geographic expansion. In Colombia, for example, episodes of mortality in primates have been documented in the Amazon and Orinoquía regions, as well as in transition zones between forests and intervened areas [42]. These events are often associated with ecological factors such as deforestation, habitat fragmentation, and land-use changes, which can modify the distribution of vectors and primates and increase opportunities for viral transmission. From a One Health perspective, the study of epizootics in NHPs enables a better understanding of the interactions among wildlife health, ecosystem health, and human health. Consequently, strengthening epizootic surveillance systems, along with vaccination and environmental monitoring strategies, represents a fundamental measure to anticipate human outbreaks and reduce the impact of YF in the region [17].

## 6. Genomic Surveillance of YF in the Context of Epizootics

Genomic surveillance of YFV has become a fundamental tool for understanding the dynamics of epizootics in NHPs and their relationship with transmission to humans. The analysis of viral sequences obtained from samples of dead or diseased primates allows the identification of circulating lineages, tracing viral dispersal routes, and detecting possible evolutionary changes associated with the virus’s adaptation to different hosts or vectors [7,56,57]. In the context of epizootics, genomic sequencing complements traditional diagnostic methods, such as RT-PCR or histopathology, by providing detailed information on the virus’s genetic diversity and its evolution over time and space. This approach has made it possible to reconstruct the recent history of YFV circulation in South America and to understand how epizootics in primates reflect viral expansion processes through forest landscapes and ecological corridors [58,59,60].

Over the past two decades, advances in next-generation sequencing (NGS) technologies have facilitated genomic surveillance during YF outbreaks and epizootics. Studies carried out during recent major events, particularly in Brazil between 2016 and 2018, showed that the virus responsible belonged to the South American genotype I and that its geographical dispersion occurred through a progressive spread from Amazonian regions to areas in the southeast of the country. The phylogenetic analysis of viral genomes obtained from NHPs and human cases enabled estimation of the rate of viral dispersion and the identification of multiple transmission events across forest fragments. These findings underscore the role of primates as amplifying reservoirs within the jungle cycle and show how genomic surveillance can help identify patterns of viral expansion before significant human outbreaks are established [61,62].

In the context of the One Health approach, the integration of genomic surveillance with epidemiological, ecological, and entomological data provides a more complete picture of YF epizootics [63]. The joint analysis of viral genomes, the distribution of susceptible primates, the presence of vectors, and environmental changes makes it possible to identify risk areas and anticipate the spread of the virus to new regions. In addition, genomic surveillance helps monitor the virus’s genetic stability and detect possible variations that could influence transmission, pathogenicity, or the effectiveness of control strategies. In this regard, strengthening regional sequencing networks and the exchange of genomic data between South American countries is essential to improve YF surveillance, optimize public health responses, and reduce the impact of future epizootics on both NHPs and human populations [63].

## 7. Importance of One Health Surveillance in YF Virus and NHPs

YF surveillance from a One Health perspective is critical to understanding and controlling the circulation of the virus in tropical ecosystems where humans, animals, and vectors interact [61]. YF is a classic zoonosis whose maintenance in nature depends on the jungle cycle between mosquitoes and NHPs, while humans serve mainly as incidental hosts. In this context, human health cannot be assessed in isolation, but must be integrated with the monitoring of wildlife and environmental factors that influence viral transmission dynamics [7,64]. Surveillance based on the One Health approach promotes collaboration between disciplines such as human medicine, veterinary medicine, epidemiology, ecology, and entomology, allowing for a more comprehensive understanding of the risk of YF virus transmission [33].

NHPs play a particularly relevant role in this approach, as many neotropical species are highly susceptible to the virus and can experience significant mortality during epizootics. Species such as howler monkeys of the genus *Alouatta* have been widely recognized as epidemiological sentinels because their mortality usually precedes the appearance of human cases in a given region. For this reason, monitoring the health and mortality of wild primates is a key tool within YF surveillance systems [9,33,40]. Timely notification of epizootics allows for the identification of areas of active viral circulation and the rapid activation of public health interventions, such as vaccination campaigns, strengthening entomological surveillance, and prevention measures targeting at-risk communities.

The implementation of One Health surveillance systems also enables the integration of information from diverse sources to improve early detection of the virus. Collecting data on primate mortality, identifying mosquito vector species, genomic analysis of the virus, and monitoring environmental changes can provide early signs of viral circulation before outbreaks occur in humans [61]. Likewise, ecological factors such as deforestation, habitat fragmentation, agricultural expansion, and climate change can alter the distribution of primates and vectors, thereby altering the virus’s transmission patterns. Integrated surveillance facilitates the identification of these changes and enables the anticipation of risk scenarios, contributing to a more timely and effective response by health authorities [52].

One Health surveillance of YF also has important implications for biodiversity conservation. Epizootics can cause significant mortality in highly susceptible primate populations, thereby affecting the structure and stability of tropical ecosystems. In this sense, protecting the health of wild primates is not only relevant to prevent transmission to humans but also to preserve biodiversity and ecological balance. Strengthening collaboration between public health institutions, environmental authorities, research centers, and local communities is essential to developing integrated and sustainable surveillance systems. In this way, the One Health approach is consolidated as a key strategy to improve the detection, prevention, and control of YF in South America [33].

Despite its conceptual strengths, implementing the One Health approach in YF surveillance is far from straightforward. Operational challenges include fragmentation between human, animal, and environmental health sectors, limited financial and technical resources in endemic regions, and inconsistencies in surveillance capacity across countries. Additionally, barriers in data sharing, lack of interoperable surveillance systems, and insufficient coordination between institutions can hinder timely detection and response. These challenges may result in delayed identification of epizootics and reduced effectiveness of preventive interventions [65,66,67].

To overcome these limitations, strengthening institutional coordination and formalizing intersectoral collaboration mechanisms are essential. The development of integrated surveillance platforms that enable real-time data exchange among the public health, veterinary, and environmental sectors can significantly enhance early warning capabilities. Investment in laboratory infrastructure, field surveillance, and workforce training is also critical, particularly in high-risk and resource-limited settings. Furthermore, community engagement and participatory surveillance, including the involvement of local populations in reporting primate mortality, can improve the sensitivity of detection. Ultimately, operationalizing One Health requires not only conceptual integration but also sustained political commitment, funding, and regional cooperation to ensure effective and sustainable YF surveillance systems [68,69,70].

## 8. Prevention Mechanisms of the YF Virus in NHP

The prevention of YF in NHPs focuses mainly on surveillance strategies, risk-control measures, and ecosystem protection, since, unlike humans, there are no systematic vaccination programs for wild populations. In most cases, primates are part of the natural jungle virus cycle. Hence, the main objective of interventions is not to eliminate viral circulation in wildlife, but to reduce the risk of transmission to humans and to detect viral activity early. In this context, the systematic monitoring of epizootics through the notification of dead or diseased primates is one of the most important tools for prevention. Early detection of mortality in highly susceptible species, such as howler monkeys (*Alouatta*), makes it possible to identify areas with active circulation of the virus and activate public health measures before outbreaks occur in humans [23,71].

Another important prevention mechanism is the management of the environment and the reduction in risk interactions among humans, primates, and vectors. The conservation of forest ecosystems and the reduction in habitat fragmentation can help maintain the natural ecological dynamics that regulate the virus’s transmission. Deforestation, agricultural expansion, and urban development in jungle areas can alter the distribution of primate and mosquito vectors, increasing the probability of contact between species and favoring the spread of the virus to new areas. In addition, human activities in tropical forest areas, such as logging, mining, or ecotourism, can increase the exposure of both humans and primates to infected vectors [50]. For this reason, environmental management and land use policies are part of indirect prevention strategies within a comprehensive approach.

Finally, community education and awareness also play a crucial role in preventing YF in NHPs. In some regions, the appearance of dead primates during epizootics has generated erroneous perceptions that lead to the persecution or elimination of these animals, under the mistaken belief that they are directly responsible for the transmission of the disease. In reality, primates act as victims of the virus and as important epidemiological sentinels [72,73]. Community education helps promote the reporting of mortality events, prevent the handling of dead animals, and foster understanding of the ecological role of primates in disease surveillance. Together, the combination of epidemiological surveillance, environmental management, and public education constitutes a fundamental strategy to prevent the impact of YF on both human and non-human primate populations within an integrated approach to public health and conservation [61].

## 9. Surveillance and Response Protocols for Suspected YF in NHP

**Figure 3:**
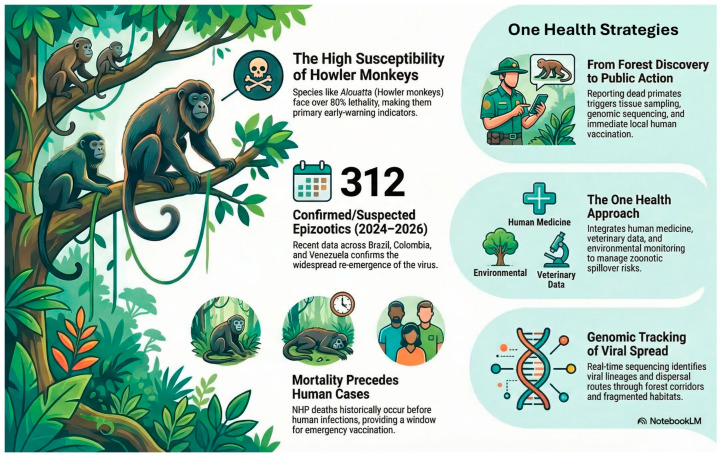
One Health Surveillance Strategies for Yellow Fever in South America. Developed with help from NotebookLM.

Several countries in South America have developed national protocols for the surveillance of epizootics in NHPs as part of their strategies for the prevention and control of YF (Figure 3). These protocols are usually coordinated by ministries of health in collaboration with environmental authorities, veterinarians, and reference laboratories, and are aligned with the recommendations of the World Health Organization and the Pan American Health Organization [17]. The basis of these systems lies in the fact that primate mortality in jungle environments frequently precedes the appearance of human cases, making these animals important epidemiological sentinels for the virus’s circulation. Countries such as Brazil, Colombia, Paraguay, and Peru have incorporated primate epizootic surveillance into their national YF surveillance systems, prioritizing early detection, diagnostic confirmation, and rapid implementation of control measures [17].

One of the most comprehensive operational frameworks has been developed by the Brazilian Ministry of Health, which developed a national guideline for the surveillance of epizootics in NHPs and entomological surveillance associated with YF. According to this protocol, any finding of sick or dead primates must be immediately reported to the health authorities through the epidemiological surveillance system. These notifications can come from local communities, park rangers, environmental authorities, researchers, or health personnel [17]. Once the event is reported, an epidemiological field investigation is initiated to confirm the event, identify the primate species involved, estimate the number of affected animals, and georeference the location where the finding occurred. This initial assessment enables the determination of whether the event is compatible with a suspected YF epizootic and guides response actions [48].

The protocols also establish standardized procedures for biological specimen collection and laboratory diagnosis. When the corpses are in suitable conditions, trained personnel collect tissue samples such as liver, spleen, and blood for analysis in specialized laboratories. Diagnosis is made using techniques such as reverse transcriptase RT-PCR, viral isolation, immunohistochemistry, or histopathological studies [74]. The reference laboratories of the national health system are responsible for confirming the presence of the YF virus. Confirmation of infection in primates is key evidence of viral circulation in the area and triggers early warning mechanisms within the epidemiological surveillance system.

Once a suspected or confirmed event has been identified, the protocols establish the immediate implementation of public health response measures. These include vaccination campaigns targeting susceptible human populations in nearby areas, strengthening entomological surveillance to detect mosquito vectors, and intensifying epidemiological surveillance to detect possible human cases. Likewise, risk communication strategies are developed to inform communities about the importance of reporting primate mortality and avoiding their handling [75]. It is critical to emphasize that primates do not transmit the disease directly to humans; rather, they serve as victims of the virus and as early indicators of its circulation. The integration of wildlife surveillance, laboratory diagnostics, and public health interventions reflects the principles of the One Health approach, which recognizes the interdependence between human, animal, and environmental health in the control of zoonotic diseases such as YF [76].

## 10. Limitations

This review has several limitations. First, as a narrative review, it depends on the availability and quality of previously published studies, which may introduce selection bias and limit the comprehensiveness of the evidence analyzed. Second, surveillance data on yellow fever (YF) epizootics in NHPs are heterogeneous across South American countries, with differences in reporting systems, diagnostic capacities, and surveillance intensity that may lead to underreporting of events. Additionally, ecological and epidemiological data remain incomplete in many regions, particularly remote forested areas. Finally, the rapid evolution of outbreaks and environmental changes may limit the temporal applicability of some observations discussed in this review.

## 11. Conclusions

Yellow fever (YF) remains a persistent and re-emerging zoonotic threat in South America, sustained primarily through the jungle transmission cycle involving mosquitoes and NHPs. As highlighted in this review, NHPs play a critical ecological and epidemiological role as amplifying hosts and early sentinels of viral circulation. Mortality events among highly susceptible species, particularly howler monkeys (*Alouatta* spp.), frequently precede human cases, underscoring the importance of epizootic surveillance as a key component of early warning systems.

Recent epizootics across several countries in the region demonstrate that YF continues to expand geographically, driven by environmental changes, forest fragmentation, human mobility, and the increasing interface between sylvatic ecosystems and human populations. These dynamics highlight the need to strengthen integrated surveillance systems that combine wildlife monitoring, entomological studies, genomic surveillance, and human epidemiological data.

The adoption of a One Health approach is therefore essential to improve preparedness and response to YF outbreaks. Enhanced regional collaboration, improved laboratory and genomic capacities, and standardized surveillance protocols will be fundamental to detecting viral circulation earlier and mitigating spillover into human populations. Ultimately, protecting both human health and wildlife populations requires sustained investment in vaccination strategies, ecological monitoring, and interdisciplinary collaboration to address the complex drivers of YF transmission in South America.

## Figures and Tables

**Table 1:** Susceptibility, mortality, and epidemiological role of selected non-human primate genera in yellow fever epizootics in the Americas.

Genus of the Non-Human Primate	Common Name	Distribution	Susceptibility to the YF Virus	Lethality in Epizootics	Epidemiological Role
*Alouatta*	Howler Monkey	Central America and much of South America	Very high	Very high (often >80%)	Key Sentinel and Amplifying Host
*Sapajus*	Robust Capuchin Jumpsuit	Brazil, Paraguay, Bolivia, Northern Argentina	Moderate	Moderate	Occasional amplifying host, sentinel
*Cebus*	Graceful Capuchin Monkey	Amazon and the tropical regions of South America	Moderate	Low to moderate	Secondary amplifier and sentinel
*Saimiri*	Squirrel Monkey	Amazonia (Brazil, Colombia, Peru, Bolivia, Venezuela)	Low to moderate	Generally low	Possible amp host with subclinical infections
*Aotus*	Night jumpsuit or night monkey	Amazon and the tropical regions of South America	Variable/poorly documented	Low	Possible Incidental Host
*Callithrix*	Tití or marmoset	Brazil (mainly Southeast and Central)	Moderate	Moderate	Sentinel in urban-wild shoots in Brazil

The qualitative categories of susceptibility and lethality are based on previously published experimental, pathological, and epidemiological evidence, including reported mortality rates during epizootics, clinical severity, and observational field data in neotropical primates. These classifications are intended to provide a comparative framework rather than precise quantitative thresholds.

**Table 2:** Clinical findings, severe disease manifestations, and complications of yellow fever infection in New World and Old World NHP.

Feature	Primates of the New World	Primates of the Old World
**Clinical Findings (General)**	Marked lethargy, progressive weakness, noticeable decrease in activity, anorexia, dehydration, and rapid deterioration of general condition; In many epizootic episodes, animals are found dead in the forest with no obvious previous signs due to rapid clinical evolution.	They frequently present subclinical infection or mild symptoms; Occasionally, lethargy, mild fever, temporary reduction in activity, and slight anorexia are observed, although many individuals remain asymptomatic and continue with apparently normal behavior.
**Manifestations of severe illness**	High susceptibility in species such as *Alouatta* and some *Atelidae*, mainly, the disease can progress rapidly, with elevated viremia, visible jaundice, severe prostration, extreme weakness, and, in some cases, neurological signs such as tremors or disorientation.	Severe disease is rare under natural conditions; however, some species may develop systemic disease in experimental infections, with fever, moderate liver involvement, and transient clinical signs.
**Complications**	Extensive hepatocellular necrosis, acute hepatic failure, internal bleeding, metabolic abnormalities, and multiorgan dysfunction; These complications are associated with high mortality rates during epizootics in highly susceptible primate populations.	Clinical complications are rare in natural populations; when they do occur, they can include moderate liver involvement, systemic inflammation, or transient febrile illness, usually with lower mortality and a higher chance of recovery.

**Table 3:** Reported epizootics from Brazil, Colombia, Bolivia, and Venezuela during the 2024–2026 YF outbreaks, according to the Pan American Health Organization, 2026. https://shiny.paho-phe.org/yellowfever/ (accessed on 1 April 2026) [47].

AL1	AL2	N	AL1	AL2	N	AL1	AL2	N
Goias	Abadia De Goias	2	Sao Paulo	Luis Antonio	6	La Paz	Abel Iturralde	1
	Firminopolis	1		Mairipora	2		Nor Yungas	1
	Goiania	2		Osasco	1		**Total**	**2**
	Guapo	1		Pedra Bela	3		**Total Bolivia**	**2**
	**Total**	**6**		Pinhalzinho	2	Aragua	Camatagua	55
Minas Gerais	Albertina	1		Pitangueiras	2		Zamora	11
	Baependi	1		Ribeirao Preto	39		San Casimiro	2
	Belo Horizonte	1		Salto	1		San Sebastian	4
	Bueno Brandao	2		Santa Rita do Passa Quatro	1		Jose Felix Ribas	1
	Camanducaia	1		Santo Antonio Do Pinhal	1		Santos Michelena	1
	Corrego do Bom Jesus	1		Serra Azul	1		**Total**	74
	Delfim Moreira	1		Serra Negra	2	Guarico	Juan German Roscio	8
	Estiva	2		Socorro	1		Ortiz	1
	Extrema	1		Valinhos	7		San Geronimo de Guayabal	2
	Ipuiuna	4		**Total**	**87**		**Total**	**11**
	Paraisopolis	1	Tocantins	Palmas	4	Portuguesa	Araure	1
	Poco Fundo	1		**Total**	**4**		Guanarito	2
	Pocos De Caldas	1		**Total Brazil**	**124**		Ospino	1
	Ponte Nova	1	Huila	Aipe	2		San Genaro de Boconoito	2
	Santa Rita De Caldas	1		Neiva	2		**Total**	**6**
	Sapucai-Mirim	2		Palermo	4	Apure	Munoz	1
	Toledo	1		**Total**	**8**		Paez	4
	Virginia	2	Meta	Villavicencio	1		**Total**	**5**
	**Total**	**25**		**Total**	**1**	Barinas	Alberto Arvelo Torrealba	1
Roraima	Alto Alegre	2	Putumayo	Mocoa	6		Sosa	1
	**Total**	**2**		Orito	2		**Total**	**2**
Sao Paulo	Amparo	2		**Total**	**8**	Carabobo	Carlos Arvelo	2
	Atibaia	1	Tolima	Ataco	10		**Total**	**2**
	Braganca Paulista	2		Chaparral	19	Cojedes	Girardot	1
	Cacapava	1		Cunday	9		Pao de San Juan Bautista	2
	Campinas	3		Planadas	6		Ezequiel Zamora	2
	Colina	1		Prado	3		Lima Blanco	2
	Cravinhos	1		Purificacion	1		**Total**	**7**
	Descalvado	1		Rioblanco	4	Lara	Simon Planas	1
	Guarulhos	1		San Antonio	5		**Total**	**1**
	Itatiba	1		Villarrica	3	Monagas	Maturin	1
	Joanopolis	3		**Total**	**60**		**Total**	**1**
	Louveira	1		**Total Colombia**	**77**		**Total Venezuela**	**109**
**Total South America**								**312**

AL, administrative level. 1, first. 2, second. N, number of epizootics.

## Data Availability

No new data were created or analyzed in this study.
